# Temporal trends in stroke admissions in Denmark 1997–2009

**DOI:** 10.1186/1471-2377-13-156

**Published:** 2013-10-30

**Authors:** Malene Nøhr Demant, Charlotte Andersson, Ole Ahlehoff, Mette Charlot, Jonas Bjerring Olesen, Anne Gjesing, Peter R Hansen, Gunnar H Gislason, Thomas Truelsen, Christian Torp-Pedersen

**Affiliations:** 1 Department of Cardiology, Copenhagen University Hospital Gentofte, Niels Andersens Vej 65, 2900 Hellerup, Denmark; 2 Department of Neurology, Herlev University Hospital, Herlev Ringvej 75, 2730 Herlev, Denmark; 3 Department of Cardiology, Aalborg University, Aalborg Hospital, Aalborg, Denmark

**Keywords:** Stroke, Temporal trends, Epidemiology

## Abstract

**Background:**

The Stroke burden is increasing in many populations where health institutions may experience more patients. We wanted to examine whether incidence rates and absolute number of hospitalized stroke patients remained stable in Denmark during a 13 years period where exposure to major stroke risk factors decreased, changes in stroke treatment was implemented, and the age of the population increased.

**Methods:**

The Danish National Patient Register was used to identify all subjects 25 years of age or above admitted with a first time stroke in Denmark from 1997–2009. Incidence rates (IRs) and age-adjusted Poisson regression analyses were used to examine trends in age-, gender- and stroke subtype (ischaemic or unspecified).

**Results:**

During the 13-year observation period there were 53.5 million person-years at risk (PY) and a total of 84,626 male and 84,705 female stroke patients were admitted to Danish hospitals. The IRs of hospitalized strokes per 1000 PY was 3.21 (95% confidence interval [CI] 3.16-3.27) in 1997, 3.85 (95% CI 3.79-3.91) in 2003 and 3.22 (95% CI 3.16-3.28) in 2009, respectively.

Incidence rate ratios of hospitalized stroke events adjusted for age in the period 2007–2009 compared to 1997–2000 were 0.89 (95% CI 0.87- 0.91) for men and 0.92 (95% CI 0.90-0.94) for women.

The incidence of hospitalized unspecified strokes decreased from 1997 to 2009 whereas there was a steep rise in incidence for hospitalization with specified ischemic stroke during this period.

**Conclusion:**

This study found a constant rate of stroke hospitalization in Denmark from 1997–2009. The overall rate of hospitalized strokes adjusted for age decreased during this period.

## Background

Stroke is a leading cause of death and acquired adult disability and it is estimated that globally each year 5.8 million people die from stroke [1]. In high income countries it has been stated that approximately 1 in 20 adults is affected by stroke and the incidence of acute cerebrovascular events (strokes and transient ischemic attack combined) exceeds the incidence of acute coronary heart disease [2]. In the recent INTERSTROKE study five risk factors including hypertension, current smoking, abdominal obesity, diet and physical activity, were associated with 80% of the global risk of stroke and interventions to reduce these factors are therefore likely to reduce the stroke incidence [3]. The observation period in this study covers a time period where smoking prevalence among Danes in general declined and treatment of hypertension and hypercholesterolemia gradually improved [4,5]. During the same period life expectancy and mean age of the population increased which could be followed by a higher absolute number of stroke events. Increasing life expectancy and mean age of the population would therefore be likely to increase the absolute number of stroke events. Different trends in stroke incidence have been reported worldwide, and it is currently not known if the stroke incidence has changed in Denmark [6-9]. Temporal changes in admission and diagnostic practices may tend to increase the overall hospitalization rates, which is a burden to health facilities even if it does not reflect an actual increase in stroke incidence rates. Therefore the aim of this study was to examine temporal trends in stroke admissions in Denmark in the period 1997–2009.

## Methods

### Data sources

All Danish residents have a unique civil registration number in the Danish Civil Registration System that enables individual level-linkage between nationwide registries. This study was based on The Danish National Patient Registry which registers all hospital discharge diagnoses since 1978. At discharge all admissions are registered by one primary and if appropriate one or more secondary diagnoses according to the International Classification of Disease, the 8^th^ revision (ICD-8) from 1978 to 1994 and the 10^th^ revision (ICD-10) from 1994.

### Population and demographics

The population demographics used in Table 1 was based on a stroke population and a control population that included all Danish citizens who were alive and older than 25 years as of January 1st 1997. The stroke population included individuals registered with a first diagnosis of stroke during the period from January 1st 1997 to December 31st 2009 and the control population included individuals with no stroke diagnosis during the period from January 1st 1997 to December 31st 2009. All individuals with previous stroke diagnosis, defined as having a primary or secondary diagnosis of stroke (all types) from January 1st 1978 to January 1st 1997, were excluded.

**Table 1:** Comorbidity ICD codes

**Comorbidity**	**ICD codes**	
Heart failure	ICD-8:	427, 428
	ICD-10:	I42, I50, I110, J819
Ischaemic heart disease	ICD-8:	411, 412, 413, 414
	ICD-10:	I20, I23, I24, I25,
Anemia	ICD-8:	280-285
	ICD-10:	D60-D69
Cancer	ICD-8:	140-209
	ICD-10:	C00-C97
Chronic obstructive pulmonary disease	ICD-8:	490, 491, 492
	ICD-10:	J42, J44
Atrial fibrillation	ICD-8:	427.4
	ICD-10:	I48
Acute myocardial infarction	ICD-8:	410
	ICD-10:	I21, I22
Peripheral arterial disease	ICD-8:	443
	ICD-10:	I70, I74
Diabetes	ICD-8:	250
	ICD-10:	E10-E14
Hypertension	ICD-8:	400, 401
	ICD-10:	I109, I119, !959, I270, I951

### Outcome

The study endpoint was stroke (all types, primary as well as secondary diagnoses) and further stratified according to subtype of stroke as unspecified stroke: 436 (ICD-8) and I64.9 (ICD-10) or ischemic stroke: 433, 434 (ICD-8) and I63 (ICD-10). Intracerebral hemorrhage (ICH): 431 (ICD-8) and I61 (ICD-10) and subarachnoidal hemorrhage (SAH): 430 (ICD-8) and I60 (ICD-10) were included in “all types of stroke”. Patients were followed until death or end of the study period i.e. December 31st 2009.

### Comorbidity

Information about preexisting conditions and past hospitalization was obtained from discharge diagnosis from The Danish National Patient Registry [10]. The comorbidity diagnoses for the stroke population were defined by presence of such diagnoses any time prior to, or on the day of the stroke. The comorbidity diagnoses for the control population were defined as whether or not they had such diagnoses any time prior to, or on July 1st 2002 i.e. after half of the study period. Codes of comorbidities are shown in Table 1.

### Statistical analysis

Categorical data are presented as numbers (percentages) and continuous variables are presented as mean with standard deviation. The number of all types of strokes, unspecified strokes and ischaemic strokes were obtained for each year from 1997–2009. Age specific incidence rates (IRs) for stroke admissions for each decade of age were calculated by dividing age-specific stroke cases by age-specific population sizes each year from the Danish Civil Registration System. The rates were stratified by gender and year of occurrence.

The study period was divided into 4 periods (1997–2000, 2001–2003, 2004–2006 and 2007–2009) and further into 6 age groups (25–45, 45–54, 55–64, 65–74, 75–84 and > 84 years). Calculations of changes in stroke incidence during the four periods were based on Poisson-regression analysis. We used a dynamic cohort population where individuals could move to a higher age-category during the follow-up period. To further adjust for the confounding effect of age each stratum was adjusted for age as a continuous variable (i.e., age in whole years). No interaction tests were done.

For all analysis a p-value of < 0.05 was considered statistically significant. The statistical packages SAS 9.2 (SAS Institute Inc., Cary NC, USA) and Stata software version 11 (StataCorp, college St. TX, USA) were used for the analysis.

## Results

### Demographic characteristics

From January 1st 1997 to December 31st 2009 167,840 admissions for first strokes were identified in the main study population of 3,601,569 persons. A total of 61,331 persons were excluded due to previous stroke diagnosis. There was equal distribution of strokes between men and women. Among all strokes 49.5% were classified as unspecific, 38.3% as ischaemic, 4.6% as SAH and 7.6% as ICH. The mean age at the time of first stroke was 71.9 (standard deviation 13.2) years.

**Figure 1:**
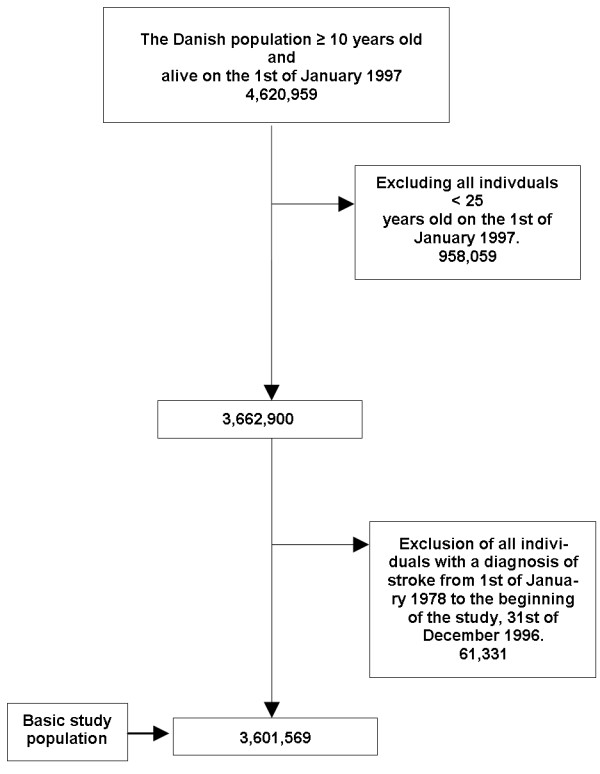
Flowchart of study population.

Figure 1 shows a flowchart of the selection of the study population and demographic characteristics are summarized in Table 2.

**Table 2:** Demographic characteristics

	**Stroke population**	**Control population**
	**N = 167840**	**N = 3433729**
Age (years)	71.9 ± 13.2	54.4 ± 16.3
Gender (% women)	50.0%	51.5%
Heart failure	19723 (11.6%)	113840 (3.3%)
Ischemic heart disease	27446 (16.4%)	154833 (4.5%)
Anemia	9231 (5.5%)	52759 (1.5%)
Cancer	25259 (15.1%)	239197 (7.0%)
COPD	11347 (6.8%)	81976 (2.4%)
AF	20960 (12.5%)	68170 (2.0%)
AMI	16865 (10.1%)	101040 (2.9%)
PAD	7778 (4.6%)	32157 (0.9%)
DM	16841 (10.0%)	80242 (2.3%)
Hypertension	33901 (20.2%)	111727 (3.3%)

COPD = chronic obstructive pulmonary disease, AF = atrial fibrillation, AMI = acute myocardial infarction, PAD = peripheral arterial disease, DM = diabetes mellitus. Data are means (standard deviations).

### Hospitalization for first time stroke 1997–2009

The total unadjusted IRs per 1000 PY were 3.21 (95% confidence interval [CI] 3.16-3.27) in 1997, 3.85 (95% CI 3.79-3.91) in 2003 and 3.22 (95% CI 3.16-3.28) in 2009.

**Figure 2:**
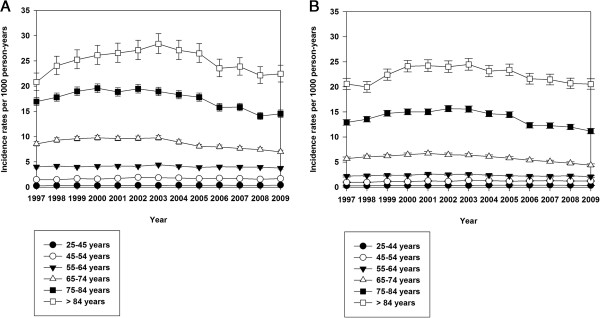
**Incidence rates, all types of stroke.** Age- and gender stratified incidence rates (with 95% confidence intervals) of hospitalization with all types of stroke in Denmark 1997–2009. **A** shows incidence rates for all types of stroke for men, **B** shows incidence rates for all types of stroke for women.

Figure 2 shows age- and gender stratified IRs for all types of strokes.

In general, stroke incidence increased with each decade of patient age.

During the 13-year observation period of 53.5 million person-years (PY) a total of 84,626 and 84,705 admissions for first stroke were registered in men and women, respectively.

**Figure 3:**
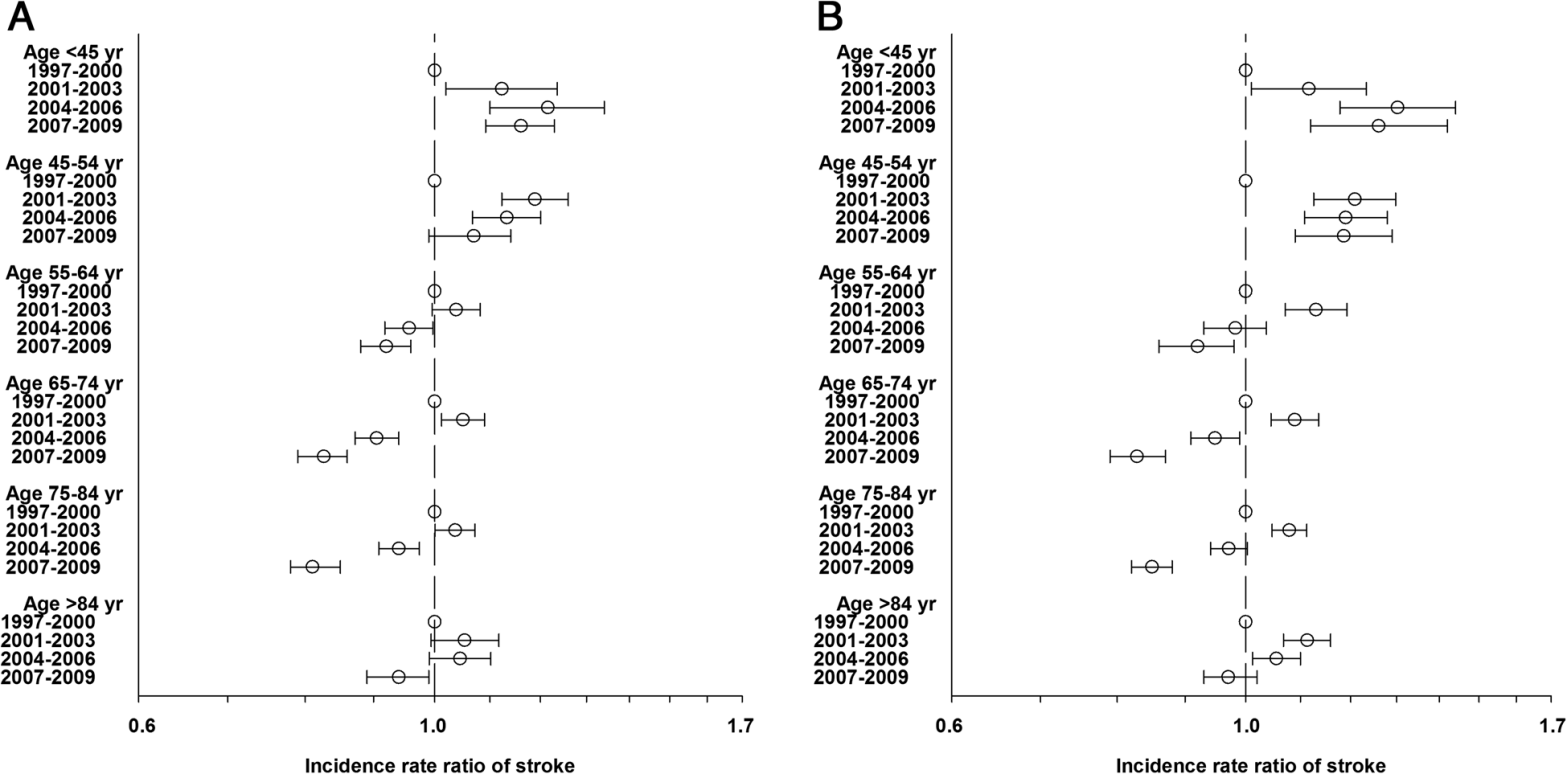
**Incidence rate ratios, all types of stroke.** Stroke incidence rate ratios (with 95% confidence interval) for all types of stroke during 4 time periods with 1997–2000 as reference. Poisson regression model. **A** shows incidence rate ratios for all types of stroke for men, **B** shows incidence rate ratios for all types of stroke for women.

Results from the Poisson regression analysis adjusted for age for all types of strokes are shown in Figure 3.

Throughout the study period a significant decrease in risk was seen in the age groups 55–64, 65–74, 75–84 and > 84 years among men, and in the age groups 55–64, 65–74 and 75–84 years among women, whereas an increase in risk was seen the age group < 45 years among men and in the age groups < 45 and 45–54 years among women, in 2007–2009 compared to 1997–2000.

Adjusted for age the incidence rate ratios (IRRs) for stroke associated with the time period 2007–2009 were 0.89 (95% CI 0.87-0.91) for men and 0.92 (95% CI 0.90-0.94) for women compared with the time period 1997–2000.

### Changes in stroke hospitalization rates according to stroke subtypes

**Figure 4:**
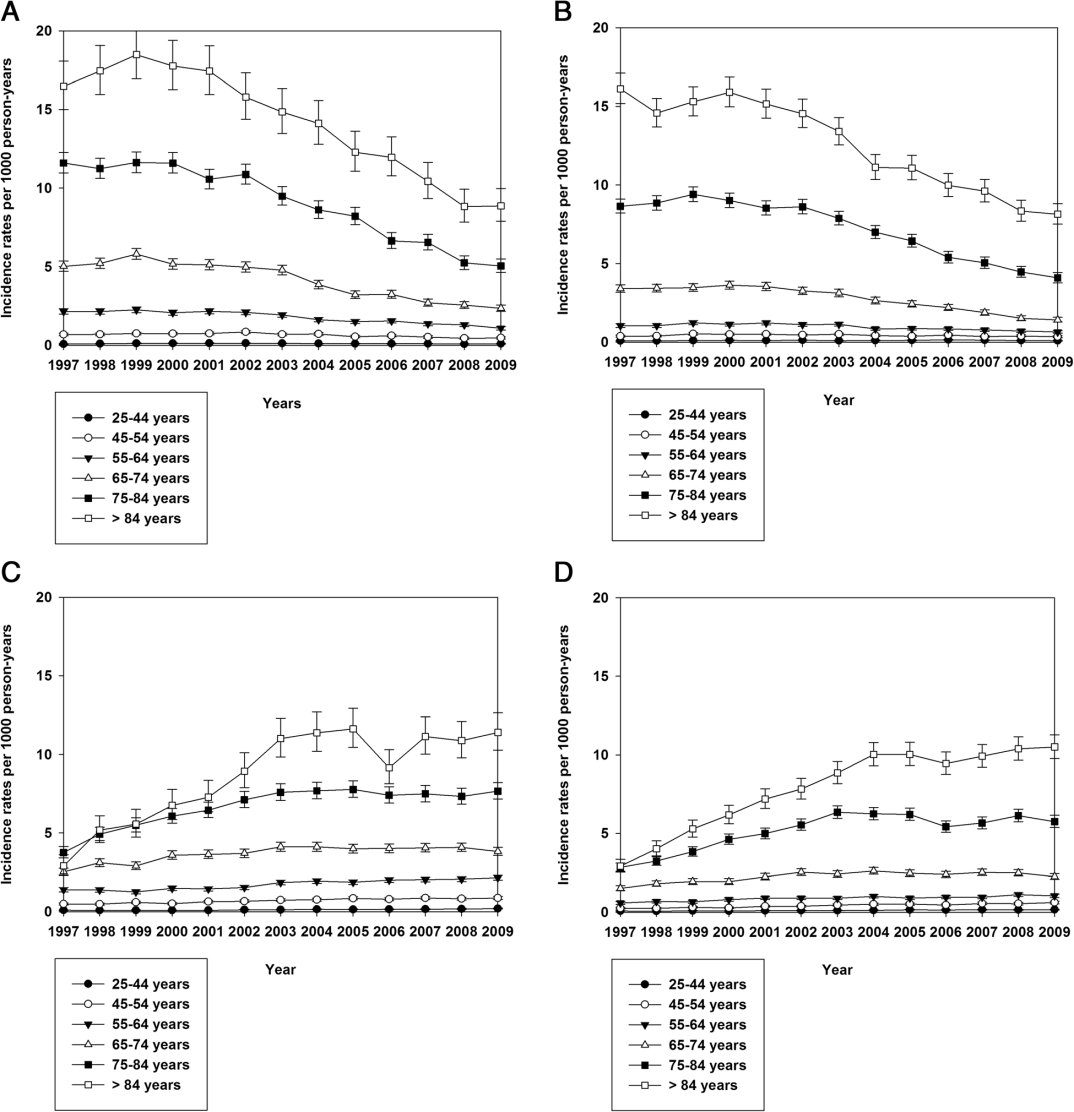
**Incidence rates, unspecified and ischemic stroke.** Age- and gender stratified incidence rates (with 95% confidence intervals) of hospitalization with unspecified and ischemic stroke in Denmark 1997–2009. **A** shows incidence rates for unspecified stroke for men, **B** shows incidence rates for unspecified stroke for women, **C** shows incidence rates for ischemic stroke for men and **D** shows incidence rates for ischemic stroke for women.

**Figure 5:**
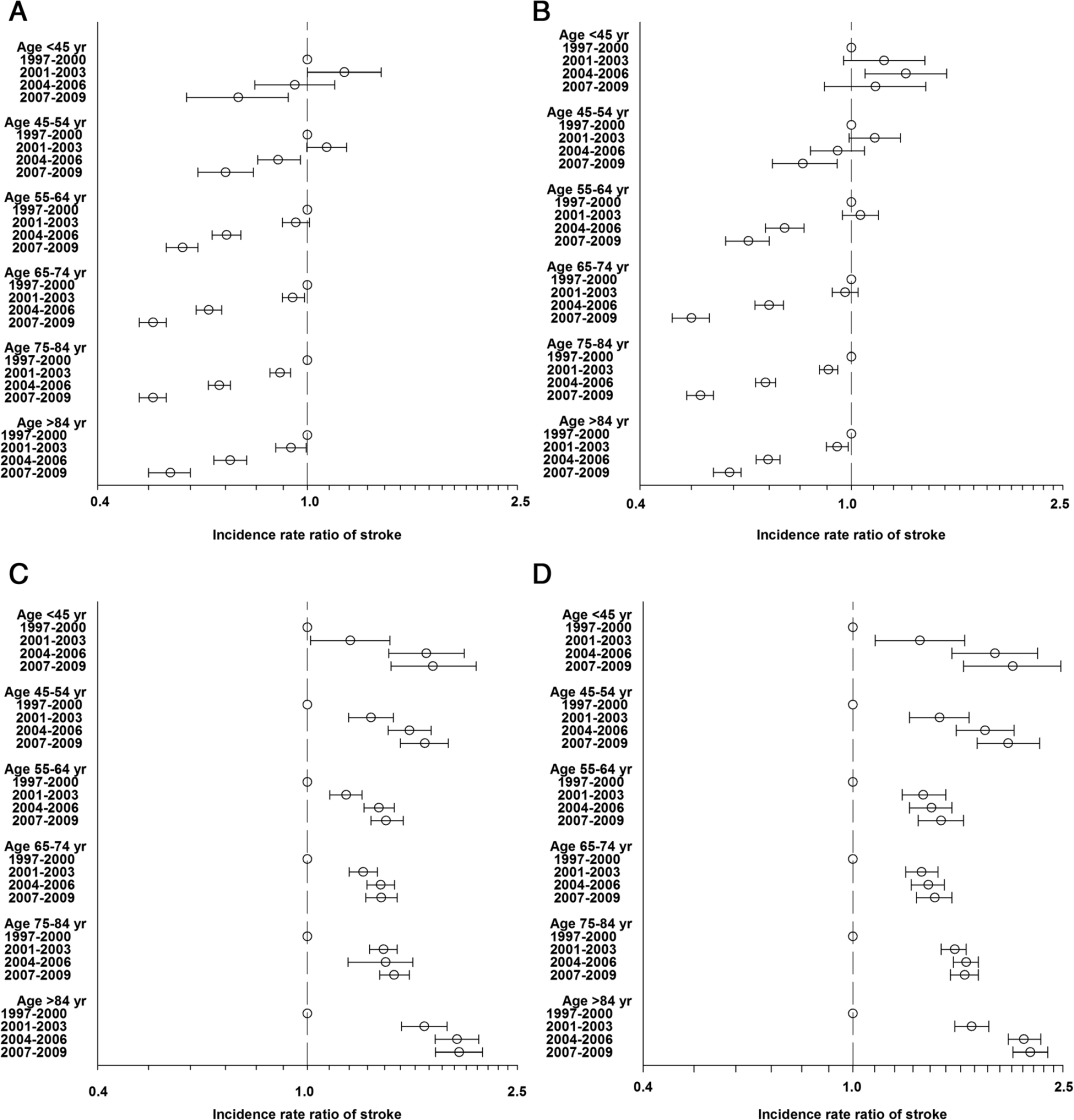
**Incidence rate ratios, unspecified and ischemic stroke.** Stroke incidence rate ratios (with 95% confidence interval) for unspecified strokes and ischemic strokes during 4 time periods with 1997–2000 as reference. Poisson regression model. **A** shows incidence rate ratios for unspecified strokes for men, **B** shows incidence rate ratios for unspecified strokes for women, **C** shows incidence rate ratios for ischemic strokes for men and **D** shows incidence rate ratios for ischaemic strokes for women.

Figure 4 shows age- and gender-stratified IRs for unspecified strokes and ischaemic strokes. Figure 5 shows stroke IRRs for men and women for unspecified strokes and ischaemic strokes.

For unspecified stroke a small increase in IRs was seen for men from 1997–1999, but hereafter a steep decline in IR occurred. This pattern was also seen for women.

The IRRs for unspecified stroke decreased from the time period 1997–2000 compared to the time period 2007–2009 in both genders in all age groups, except in the age group < 45 years in women.

For ischaemic strokes a steep rise in IR was seen for both men and women from 1997 to 2009. For all age groups and both genders a significant increase in IRRs for ischemic stroke were seen for all time periods compared to 1997–2000.

## Discussion

This nationwide study showed that in the Danish population there was a constant rate of hospitalized stroke events of 3.2/1000 PY from 1997–2009. A decrease in the stroke hospitalization rate was seen when adjusted for age. IRs for hospitalized ischaemic stroke increased during the study period whereas the IRs for hospitalized unspecified stroke decreased. Compared to the control group the stroke population was generally older and suffered from more comorbid conditions.

Different trends in stroke incidence have been reported previously in different countries. In a systematic review of 56 population-based studies from 2009, based on multiple sources of information, the authors reported a 42% decrease in stroke incidence in high-income countries, and a more than 100% increase in low to middle income countries [7]. The World Health Organization (WHO) Monitoring Trends And Determinants In Cardiovascular Disease (MONICA) stroke project did not report a definite change in stroke incidence over time [9]. Multinational comparison is possible with appropriate data quality although caution is required, as stated by others [11]. The Copenhagen City Heart Study, a prospective study based on discharge diagnosis as well as supplementary patient files, found that stroke incidence from 1976–1993 remained stable among men aged 45–64 years and women aged 45–84 years, whereas a statistically significant decrease was found in men aged 65–84 years [12]. Similarly, another Danish prospective study based on clinical stroke diagnoses evaluated by a trained neurologist, in the community of Frederiksberg, Copenhagen, found an increase of 42% in stroke incidence for men aged 65–84 years from 1972–1974 to 1989–1990 [13]. There were no changes in IRs for women. In Lund, Sweden, in a study involving retrospective screening of medical journals, increasing IRs were found, mainly confined to people < 75 years of age [14]. In a recent cohort study from Rotterdam, the Netherlands, based on stroke diagnosis from general practitioners as well as hospital files reviewed by a stroke neurologist, the authors found that IRs from 1990–2008 increased in men, but not in women [15].

The observation period in this study covered a time period where smoking prevalence among Danes in general declined and treatment of hypertension and hypercholesterolemia gradually improved [4,5]. On the other hand the incidence of atrial fibrillation (AF) is likely to have increased, partly due to increasing population size as well as a larger proportion of elderly [16,17]. AF is an important risk factor for stroke and is estimated to be associated with approximately 1/5 of ischaemic stroke events, which could increase in the absolute number of stroke events due to the rise in AF incidence [18,19]. Furthermore, it has been suggested that an increase in stroke occurrence might be due to increased survival after ischaemic heart disease [20]. Increased focus on and awareness of stroke symptoms in both the general population and in the health care system may lead to improved detection of less-severe strokes and which could increase the number of patients discharged with a diagnosis of stroke [21]. Introduction of thrombolytic treatment given to carefully selected patients may have further increased the focus on early recognition and diagnosis of stroke events, as the chances of successful outcome decrease by the minute after onset of stroke symptoms [22,23]. This may also have resulted in a tendency in the current era to perform cerebral CT scanning of patients with milder cases of strokes, which would not have been diagnosed before. As such, the fact that the number of patients diagnosed with ischaemic stroke increased over time in our study may be explained, in part, by increased use of cerebral imaging procedures. During the later years more hospital units have gained access to CT and MRI scanners that make it more likely that the stroke diagnosis has become more specific. Therefore, the increasing IRs of ischaemic strokes observed in our study is more likely to reflect the implementation of scanners in the Danish health care system than an actual increase in actual ischemic stroke rates. This notion may also explain why the IRs of patients admitted with unspecified stroke decreased in the observed time period. The decreasing rates may again reflect that more and more patients are scanned and may therefore receive a more exact stroke diagnosis.

Recent WHO estimates based on reviews of studies including more than 20 million subjects, project that the number of strokes in European countries is likely to increase from 1,1 million per year in 2000 to 1,5 million per year in 2025 [24]. Moreover, the median age in these populations is envisioned to rise from 37.7 years in 2000 to 47.7 years in 2050 along with a decrease in the total number of Europeans. Overall, these projections suggest that the absolute number of strokes will increase in future decades, whereas the number of younger adults will decrease. This development is likely to put further burden on the welfare society and health care systems in European countries [25,26].

This study reflects the number of patients discharged from hospital and every patient admitted for a stroke calls for hospital facilities as well as urgent attention from physicians and nurses. The large group of patients in need of assistance and rehabilitation is also a significant expense for the health care system as half of all stroke patients suffer from permanent ill-effects and almost one-out-of-four acquire disabilities to such an extent that help to everyday activities is necessary [27]. Furthermore there is an additional cost due to the decreased employability and early retirement of stroke patients, as well as the fact that one third of all stroke patients suffer from depression [28]. The socioeconomic importance of the disease cannot be underestimated.

### Strengths and limitations

This was a retrospective register based study only including hospitalized cases of strokes. Caution is therefore required when making conclusions regarding the true IRs of strokes in Denmark as several factors may impair the use of hospital discharge diagnoses for direct disease analysis. Registers are vulnerable to changes in registration practices, which may alter the sensitivity and specificity of the diagnosis. For example, studies have shown that a stroke discharge diagnosis from a specialized neurology unit had a higher positive predictive value than a stroke diagnosis from emergency rooms [29]. This may reflect a greater expertise, routine and interest in the treatment and diagnosis of stroke patients in specialized units, along with the fact that emergency rooms often have much less time before the patient is transferred to other units, and may not have had time to evaluate neuroimaging results. The validation of stroke discharge diagnosis in The Danish National Patient Registry has also been shown to differ substantially among stroke subtypes, and the register appears to generally overestimate the number of strokes [29,30]. Indeed, while two studies from 2001 and 2007 showed positive predictive values of 88% and 98% for the ischaemic stroke diagnosis, respectively, the majority (60%) of unspecified strokes was identified in cases diagnosed with ischaemic strokes and 5% of unspecified strokes were identified in cases diagnosed with ICH [29,30]. In addition the Danish National Patient Registry contains no information on smoking status and body mass index and no adjustments were therefore made for these stroke risk factors.

## Conclusion

This study found a constant rate of stroke hospitalization from 1997–2009. The overall rate of stroke hospitalized stroke events adjusted for age was reduced during the study period. This study was the first to investigate trends in patients discharged with a first-time stroke diagnosis in Denmark. Effective preventive and treatment strategies are very much needed, as the burden of stroke will increase as the elderly segment of the population increases.

## Competing interests

The authors declare that they have no competing interests.

## Authors’ contributions

MD: acquired and interpreted data, performed statistical analysis and prepared the manuscript CA, OA, MC, AG and JBO: participated in drafting the manuscript, performed statistical analysis, interpreted the results, and critically revised the intellectual content of the article. PRH, GG, TT and CTP: participated in study concept and design, the acquisition, analysis and interpretation of the data, performed statistical analysis and critically revised the intellectual content of the article. All authors have read and approved the final manuscript.

## Pre-publication history

The pre-publication history for this paper can be accessed here:

http://www.biomedcentral.com/1471-2377/13/156/prepub
